# Dry-Coated Graphite onto Sandpaper for Triboelectric Nanogenerator as an Active Power Source for Portable Electronics

**DOI:** 10.3390/nano9111585

**Published:** 2019-11-08

**Authors:** Smitha Ankanahalli Shankaregowda, Rumana Farheen Sagade Muktar Ahmed, Yu Liu, Chandrashekar Bananakere Nanjegowda, Xing Cheng, Srikantaswamy Shivanna, Seeram Ramakrishna, Zhenfei Yu, Xiang Zhang, Krishnaveni Sannathammegowda

**Affiliations:** 1 Department of Electronics, Yuvaraja’s College, University of Mysore, Mysuru 570005, Karnataka, India; smithamtech@gmail.com; 2 Department of Studies in Physics, University of Mysore, Mysuru 570006, Karnataka, India; rumanafarheen1993@gmail.com; 3 Shenzhen Key Laboratory of Nanoimprint Technology, Department of Materials Science and Engineering, Southern University of Science and Technology (SUSTech), Shenzhen 518055, China; liuy3@mail.sustech.edu.cn (Y.L.); chandrashekar@sustech.edu.cn (C.B.N.); chengx@sustc.edu.cn (X.C.); 4 Guangdong Provincial Key Laboratory of Energy Materials for Electric Power, Shenzhen 518055, China; 5 Centre for Materials Science and Technology, Vijnana Bhavan, University of Mysore, Manasagangotri, Mysore 570006, India; 6 Department of Mechanical Engineering, National University of Singapore, Singapore 117576, Singapore; seeram@nus.edu.sg; 7 School of Materials Science and Engineering, Beijing Institute of Technology, Beijing 100081, China; yuzhenfei@bit.edu.cn; 8 School of Mechanical Engineering, Beijing Institute of Technology, Beijing 100081, China

**Keywords:** graphite, sandpaper, flexible dry electrode, biomechanical sensor

## Abstract

Developing an eco-friendly, flexible and recyclable micro-structured dry electrode for sustainable life is essential. In this work, we have developed irregular, micro-structured sandpaper coated with graphite powder as an electrode for developing a simple, low-cost, contact-separation mode graphite-coated sandpaper-based triboelectric nanogenerator (GS-TENG) as a self-powered device and biomechanical sensor. The as-fabricated GS-TENG is a dielectric-conductor model. It is made up of a bottom layer with polytetrafluoroethylene (PTFE) as a triboelectric layer, which is attached onto a graphite-coated sandpaper-based electrode and a top layer with aluminum as another triboelectric layer as well as an electrode. The forward and reverse open-circuit voltages reach upto ~33.8 V and ~36.62 V respectively, and the forward and reverse short-circuit currents are ~2.16 µA and ~2.17µA, respectively. The output generated by GS-TENG can power 120 blue light-emitting diodes connected in series, liquid crystal display and can charge commercial capacitors along with the rectifier circuit. The capacitor of 22 µF is charged upto 5 V and is sufficient to drive digital watch as wearable electronics. Moreover, the device can track signals generated by human motion, hence it scavenges biomechanical energy. Thus, GS-TENG facilitates large-scale fabrication and has potential for future applications in wearable and portable devices.

## 1. Introduction

Nowadays, wearable and portable smart electronic devices, such as flexible display [1], wearable sensors [2,3,4], flexible batteries [1,5,6] and sustainable power sources [7,8,9,10], have gained much attention due to their potential applications. Most of these devices depend on electricity and rechargeable batteries, to maintain the continuous and independent working of electronic devices, and the desire to replace batteries has raised great interest for researchers to find alternative power sources. Since most commonly used conventional batteries as a power source limits their applications due to their bulk size, monitoring limited life-time remains as a technological threat and has to be replaced [11]. Green energy harvesting from the living environment and utilizing it effectively to power all possible electronic devices has become an urgent need [12,13,14]. Therefore, a new power source with miniaturization, which is flexible, sustainable and maintenance-free, is greatly needed and could be used widely in health monitoring [15,16], wearable sensors [2,3], nano-robotics [17], remote environment sensors [18] and actuators [19]. As a solution, a triboelectric nanogenerator (TENG) has recently been invented as a promising power source, which provides a compelling route to convert energy from mechanical form to electrical, based on the conjunction of two physical phenomena, triboelectrification (also called contact-electrification) and static-induction [20]. The human motion-related mechanical energy such as hand tapping, tilting neck, finger motion, wrist movement, bending knees, stamping foot etc., can be effectively harvested by TENGs, converting the bio-mechanical energy into electrical energy [21,22]. Harvesting energy from flexible wearable TENGs by human body movements shows its unique advantage as a self-powered active motion sensor. These electronic devices are based on flexible polymers with suitable flexible electrodes, which have manufacturability, durability and capability of integration with other technologies. Electrodes are extremely essential components for TENG for its active functioning. Commonly, plastic-based electrodes are integrated with TENGs, as they are highly flexible, light-weight and convenient [23,24]. However, they are not suitable to work in high-temperature environments due to deformation and high thermal coefficient of expansion [25], which hinders their potential application. Most commonly, as an alternative to plastic electrodes, cellulose paper-based electrodes are found to be reliable and facile for the fabrication of TENGs due to its flexibility and fiber-like surface structures, which provide a larger surface area to bind conductive materials [26,27,28]. So far, solution-based electrodes by wet technique [29], embedding silver nanowires on paper through the infiltration technique [30], stacking metal films on paper [31] and the penciling paper technique [32] are adopted to make conductive paper. In all these methods, fiber structures on the surface of paper play a vital role to adhere any of the conductive materials onto it. Despite the considerable progress made in paper-based electrodes/TENG, coating-conducting materials on paper are usually pasting or wet solutions, which are likely to degrade and are not durable for long-term use. Therefore, it is desirable to find another strategy to develop flexible, corrosion-free, low-cost, metal-free, durable and robust electrodes/TENGs with cost-effective large-scale production.

Sandpaper can be a promising candidate for electrode fabrication due to economical, flexible, durable and earth benevolent. Sandpaper is assessed according to the grit size (number of holes per linear inch in a sieve screen) [33]. Also, it inherently creates nano-micro scale roughness levels [34,35]. In comparison with traditional substrates for the TENG, it cut down the time-consuming and additional expensive fabrication procedures to achieve nano-micro roughness to enhance the effective contact area of the TENG. The significance of a sandpaper-based electrode depends on the risk-free manufacturing procedure, recyclable or disposable and economical, along with the necessary requirements. 

In this work, we demonstrate a flexible, contact-separation mode graphite-coated sandpaper-based triboelectric nanogenerator (GS-TENG) by a simple fabrication technique. In the GS-TENG, the bottom layer is made of polytetrafluoroethylene (PTFE) as a friction layer, sandpaper-based electrode, and the top layer of the GS-TENG consists of aluminum (Al) as a friction layer as well as an electrode. The sandpaper-based electrode is prepared by coating graphite powder on to the sandpaper using a brush. To ensure good conductivity and mechanical durability, the sandpaper-based electrode along with PTFE is subjected to a bending test. Under repeated cycles of bending and releasing, the resistance of the electrode is ~12.5 kΩ, remains almost constant throughout the bending cycles for more than 30 h. The as-fabricated GS-TENG is subjected to forward and reverse polarity tests for the validity of signal generation, the peak-to-peak forward and reverse open-circuit voltages up to ~33.8 V and ~36.62 V respectively, and the peak-to-peak forward and reverse short-circuit currents up to ~2.16 µA and ~2.17µA respectively, were obtained under external vibration at frequency 4 Hz. The energy conversion efficiency is found to be ~7.7% and the maximum peak power density reached upto ~0.94 µW·cm^−2^ at a load resistance of 30 MΩ. This is sufficient to light-up more than 120 blue light-emitting diodes (LEDs) connected in series and to power a liquid crystal display (LCD). In addition, by conducting a charging ability test of GS-TENG by charging several capacitors with different capacitance by hand tapping, the stored energy is utilized to power a smart watch. Furthermore, GS-TENG can be used as a self-powered human motion sensor, which can track signals generated by the human body, such as finger tapping, wrist movement, hand tapping and foot stepping. Thus, as-fabricated GS-TENG facilitates robust, low-cost, and ease of fabrication, which illuminates its potential for future applications as a sustainable power source and for wearable motion sensors.

## 2. Materials and Methods

### 2.1. Preparation of Sandpaper-Based Electrode

**Figure 1:**
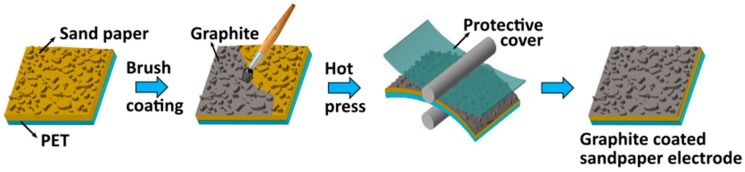
Schematic of the complete fabrication process of the graphite-coated sandpaper electrode.

Graphite powder was purchased from BENNO (Model No. AG1299) and sandpaper with grit size 400 was purchased from softflex 991A (made in Germany). Initially, the sandpaper with the size 45 × 45 mm^2^ is attached to the commercially available polyethylene terephthalate (PET) with the size 45 × 45 mm^2^, which brings good mechanical durability. A pinch (~1 mg) of as-purchased graphite powder is coated on to the sandpaper/PET using a commercially available nylon hair acrylic paint brush. Then the graphite-coated sandpaper/PET is hot-pressed using a roll-to-roll lamination machine (Model no. YE381L SOONYE^®^, China) to ensure firm coating of graphite powder onto the sandpaper. To avoid the adhesion of graphite powder onto the rollers, a thin film of PET as a protective cover is used and finally, the protective cover is removed from the graphite-coated sandpaper/PET and the same is used as an electrode for further device fabrication. The complete preparation process of the graphite-coated sandpaper electrode is schematically shown in Figure 1.

### 2.2. Fabrication of Triboelectric Nanogenerator (TENG)

**Figure 2:**
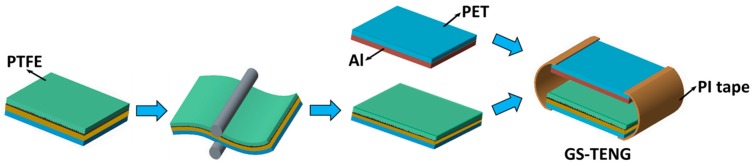
Schematic illustration of the fabrication process of the graphite-coated sandpaper-based triboelectric nanogenerator (GS-TENG).

The schematic illustration of fabricating GS-TENG is shown in Figure 2. The GS-TENG is based on the conductor-dielectric model with contact-separation mode. The bottom layer is made by assembling PTFE film tape (YLH-7018M) on to the sandpaper-based electrode (as demonstrated in Figure 1). The graphite-coated sandpaper electrode is used as a passive electrode. After dry-coating of graphite onto sandpaper, it is encapsulated with PTFE adhesive tape to firmly hold the graphite powder with sandpaper. As PTFE is a good triboelectric material due to its high tribo-negative nature, according to the triboelectric series [36], and its low cost, high mechanical durability and high output efficiency characteristics makes it a suitable candidate for TENG. Then, the stacked structure is subjected to the roll-to-roll press to ensure firm adhesion of graphite powder onto the sandpaper. A thin film of Al with the thickness of 0.13 mm is attached on to the PET film (0.13 mm), forming the top layer of GS-TENG. Since Al is one of the suitable metals due to its high conductivity, ductility and its low cost [37], it is used as a functional electrode as well as triboelectric material for TENG. Both top and bottom layers are connected using polyimide (PI) tape, as illustrated in Figure 2, which easily helps in effective contact and separation, thus completing the fabrication of GS-TENG.

### 2.3. Characterization and Electrical Measurement

The surface characterization was done with scanning electron microscopy (SEM) (ZEISS-Merlin, Oberkochen, Germany). The contact angle measurement was performed by using a contact angle tester (AST products Inc., Billerica, USA). The short-circuit current and open-circuit voltage were measured by the Stanford low-noise current preamplifier (Model SR570, Stanford research system, Sunnyvale, CA, USA) and electrometer (Keithley 6514 System Electrometer, Beaverton, OR, USA), respectively. An electrodynamic vibration exciter, Bruel and kjaer (Model No 4808, Bruel & Kjaer Co., Naerum, Denmark), was employed as an external vibration source with sinusoidal output.

### 2.4. Comsol Simulation

**Figure 3:**
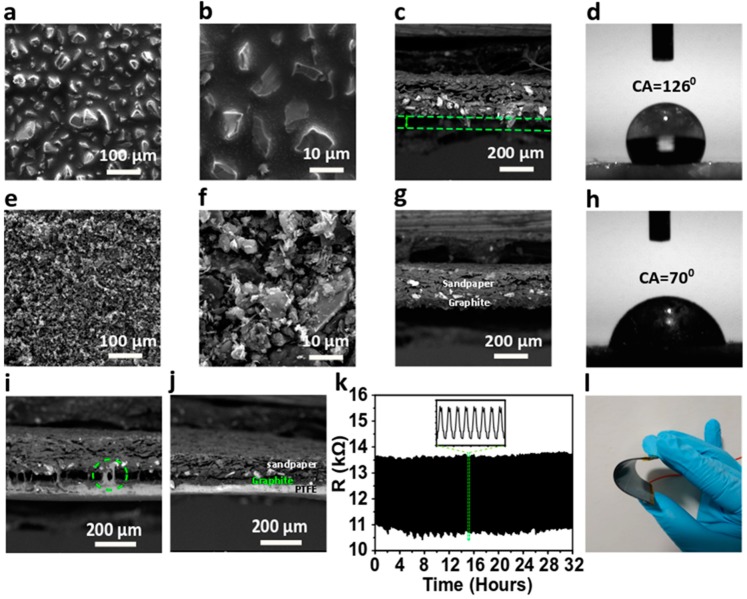
Surface morphological Characterization, wettability and bending test. Scanning electron microscope (SEM) images of bare sandpaper at (**a**) Higher magnification, (**b**) Lower magnification, (**c**) Lateral view and (**d**) Optical image of contact angle showing hydrophobic nature. SEM images of graphite-coated sandpaper at (**e**) Higher magnification, (**f**) Lower magnification, (**g**) Lateral view and (**h**) Optical image of contact angle showing hydrophilic nature. SEM images of graphite-coated sandpaper with polytetrafluoroethylene (PTFE) film (**i**) before and (**j**) after roll-to-roll hot pressing. (**k**) Bending test of graphite-coated sandpaper/polyethylene terephthalate (PET) with PTFE film tape. (**l**) Optical image of GS-TENG showing flexibility.

For as-fabricated GS-TENG, finite element analysis was carried out to obtain better quantitative understanding of the working mechanism. COMSOL Multiphysics (4.0, COMSOL, Inc.) simulation software was employed to calculate the potential distribution between the graphite-coated sandpaper electrode and Al. The simulated conductor-dielectric model was based on Al and PTFE with the dimensions 45 × 45 mm^2^, as shown in Figure 3e. The thickness of the Al layer and PTFE film was set to be 0.13 mm and 0.06 mm, respectively. The Al surface and the PTFE surface were assumed to be filled with charge density of +20 µC·cm^−2^ and –20 µC·cm^−2^.

## 3. Results and Discussion

### 3.1. Characterizations of the Graphite-Coated Sandpaper-Based Triboelectric Nanogenerator (GS-TENG)

Micro/nano-scale of silicon carbide particles as abrasives embedded on sandpaper provides an irregular surface texture. Graphite powder is uniformly spread on to the micro/nano-meshes in the sandpaper using a nylon hair acrylic paint brush. Then, the graphite-coated sandpaper/PET is hot-pressed using the roll-to-roll technique and finally, graphite-coated sandpaper/PET is used as an electrode for further device fabrication. The more detailed fabrication process is discussed in Section 2.1. To study the surface morphology of the sandpaper-based electrode, the scanning electron microscopic (SEM) images at each stage of electrode fabrication were taken. A typical SEM image of bare sandpaper with the grit size 400, as depicted in Figure 3a at lower and higher magnification (Figure 3b), shows a high density of non-uniform micro/nano-meshes. The vertical cross-sectional SEM reveals sharp edges of micro/nano-meshes’ groves (Figure 3c), revealing sandpaper roughness, there is a micro gap between the sandpaper base and groves tips, as indicated from the green dashed lines. This space is well utilized to coat conductive graphite powder, so that amorphous graphite powder evenly fills the textured surface of sandpaper. Moreover, a contact angle (CA) of 126° (Figure 3d) is formed when a water drop is placed on top of the sandpaper, owing to the presence of micro/nano-meshes, which can withstand in a harsh environment. The SEM image of graphite-coated sandpaper is shown in Figure 3e. It can be seen that the micro/nano-groves are completely filled after graphite coating, which confirms that the graphite powder is uniformly distributed on the surface of sandpaper filling the gaps between micro/nano-groves (at higher magnification in Figure 3f). The cross-sectional view of graphite-coated sandpaper, as shown in Figure 3g, depicts adhesion of graphite powder on sandpaper after being subjected to roll-to-roll hot pressing. Further, Figure 3h shows the wettability test through (CA)measurement of graphite-coated sandpaper. In general, graphite powder is hydrophobic [38], but after coating on sandpaper, the contact angle measured is 70°. This is primarily attributed to the fact that the meshes in the sandpaper lead to the asymmetrical (bumpy) layer of graphite coverage, which can be seen in the highly magnified SEM image (Figure 3f). Thus, showing the virtual hydrophilic nature of graphite-coated sandpaper confirms that micro/nano-meshes were completely occupied with the graphite layer.

Further, PTFE film tape is stacked on the as-prepared sandpaper-based electrode, as schematically shown in Figure 2. The cross-sectional view of the SEM image (Figure 3i) shows that the binding tendency of PTFE with the coated graphite on sandpaper is weak. Noticeably, there is a wide micro-gap of a few micrometers approximately between PTFE and graphite-coated sandpaper, which is due to the fact that the uneven grooves cannot stick well with PTFE. Also, a layer of graphite adhered to the PTFE fails to extensive binding with sandpaper (indicated with a circle). Hence, the stacked structure undergoes the roll-to-roll press to ensure good conductivity, as shown in the schematic in Figure 2. The stack thickness of ~300 µm is passed between the rollers of 200 µm space, due to pressing pressure, each layer is firmly bound, which is shown in the cross-sectional SEM image (Figure 3j), forming a bottom layer of GS-TENG. Furthermore, the bottom layer of the GS-TENG (PTFE/graphite-coated sandpaper/PET) is subjected to a mechanical deformation test by measuring resistance under the repeated bending and releasing cycle of PTFE/graphite-coated sandpaper/PET. The resistance of the graphite-coated sandpaper electrode obtained is ~12.5 kΩ, which remained almost constant throughout the bending cycles for more than 30 h, as shown in Figure 3k, which ensures good conductivity and mechanical stability of the electrode. Supplementary Table S1 summarizes the unique characteristics of the graphite-coated sandpaper electrode over other metallic-based electrodes. With good conducting properties, along with being economical, Al is preferred for the fabrication of GS-TENG. A thin film of Al with a thickness of 50 µm is attached to PET, forming the upper layer. Both the upper and lower layers are mounted on a PET substrate, as PET enhances the flexibility and durability of the device. The Al/PET upper layer and lower layer PTFE/graphite-coated sandpaper/PET are assembled to form GS-TENG, with an area of 45 × 45 mm^2^, as illustrated in Figure 2 and in optical image (Figure 3l). The commercial availability, low-cost and adaptability of the materials utilized in the fabrication process of the device provide a route for large-scale industrialization.

### 3.2. Working Principle of TENG

**Figure 4:**
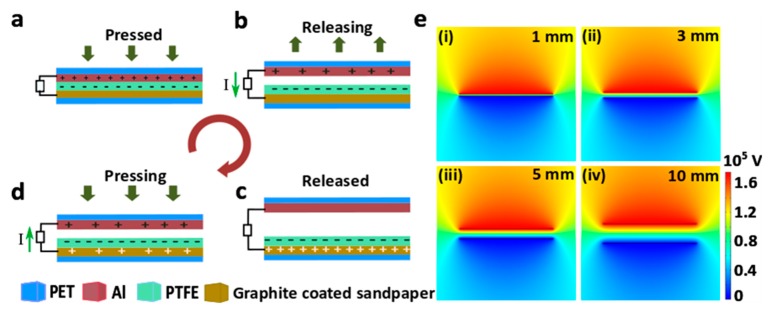
Schematic illustration of working mechanism of the GS-TENG. (**a**) Initially, the upper aluminum (Al) electrode is fully in contact with the PTFE surface. (**b**) The release of the external force causes electrons to flow from the upper Al electrode to the bottom sandpaper/graphite electrode through an external circuit. (**c**) The equilibrium state. (**d**) Electrons flow back due to the reapplied external force. (**e**) Comsol simulation results of GS-TENG at different separation distances between PTFE and Al.

The working mechanism of GS-TENG is shown in Figure 4. The conductor-dielectric model consists of Al (conductor) as a tribo-positive layer and PTFE (dielectric) as a tribo-negative layer, according to triboelectric series [36]. When the Al layer is in complete contact with the PTFE, Al and PTFE surfaces are rendered with positive and negative charges respectively, due to triboelectrification, as shown in Figure 4a. As the layers are separated, potential difference-developed drifts free electrons from the Al electrode to the graphite-coated sandpaper electrode through an external load (Figure 4b). Now, when both the layers are separated to the maximum separation distance (Figure 4c), electrostatic equilibrium occurs between the electrodes, and hence, no charges flow. When both the layers are contacted again by an external pressing force, charges flow back to the Al layer (Figure 4d). Therefore, an alternate current is generated by repeated contact and separation. Figure 4e(i–iv) shows the comsol simulation results of the potential distribution in GS-TENG for different separation distance of 1 mm, 3 mm, 5 mm and 10 mm. The electric potential on the Al surface reaches 10^5^ V when they are separated to 10 mm. The simulation results of the device show that, as the separation distance between the Al and PTFE layer increases, the potential difference also increases and reaches maximum.

### 3.3. Performance of GS-TENG

**Figure 5:**
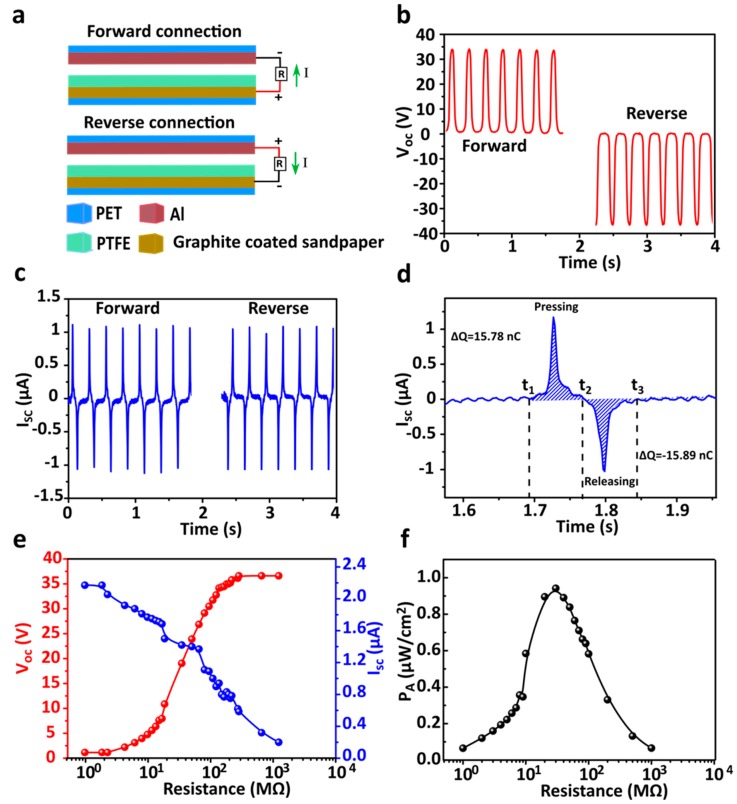
Output performance of the GS-TENG**.** (**a**) Schematic of the forward and reverse connection. (**b**) Open-circuit voltage in the forward and reverse connection. (**c**) Short-circuit current in the forward and reverse connection, respectively. (**d**) One complete cycle of the short-circuit current with the calculated total amount of charges transferred in each peak. (**e**) Plot of open-circuit voltage and short-circuit current versus external load resistance. (**f**) Output power density versus load resistance of GS-TENG.

To investigate the GS-TENGs triboelectric output performance, with an effective contact area of 45 × 45 mm^2^, contact was made periodically and separated under external vibrator excitation at a frequency of 4 Hz and constant amplitude. Initially, we conducted a switching polarity test to know the output signals truly generated from TENG. The ability of electrons to flow from the graphite-coated sandpaper electrode to Al due to the potential difference developed during contact and separation is explained in the working mechanism in detail. By connecting the positive terminal of the probe to the sandpaper-based electrode and the negative terminal to the Al electrode as forward connection mode (Figure 5a), the peak value open-circuit voltage and the short-circuit current were measured at ~33.8 V and ~2.16 µA, respectively. Then, the probe connections were switched reversibly to change the polarity as reverse convention mode (Figure 5b & c). The electrons flow from Al to the graphite-coated sandpaper electrode. Open-circuit voltage and short-circuit currents of peak values were measured at~36.62 V and ~2.17 µA, respectively. As evidence for the switching polarity, the output performance of the as-fabricated GS-TENG validates the switching polarity test and results were significant in both directions. The amount of charge transfer between electrodes with reference to the positive current peak of 15.78 nC was obtained, as shown in Figure 5d. The output voltage current and output current of GS-TENG was measured by varying load resistance ranging from 10 to 100 MΩ.

The voltage curve increases with the increasing load resistance and the current decreases with the increasing load resistance, as shown in Figure 5e. The output power density is obtained using the equation, *P* = *I*^2^*R/A*, where, *I* is the current value at external load resistance *R* and *A* is the contact area of the TENG, respectively. Initially, power density was increased and reached the maximum at matched load resistance and further decreased as external load resistance increased, as shown in Figure 5f. When the external load resistance matches the internal impedance of GS-TENG, the power density reaches maximum value. According to the maximum power transfer theorem [39], the maximum peak power density obtained is ~0.94 µW·cm^−2^ at optimal load resistance 30 MΩ, which is sufficient to drive low-power consumption electronics, proliferating the applications range of GS-TENG. In addition, energy conversion efficiency of the GS-TENG with 7.7% was calculated [40] (Supplementary Note: 1). The conducting stability of the GS-TENG is examined by using the graphite-coated sandpaper electrode with different grit sizes of the sandpaper (400, 600, 800, 1000, 1200, 1500, 2000). Irrespective of grit size of the sand paper, GS-TENG performed consistent output voltage, as shown in Supplementary Figure S1.

### 3.4. Application of GS-TENG for Energy Harvesting and Self-Powered Devices

**Figure 6:**
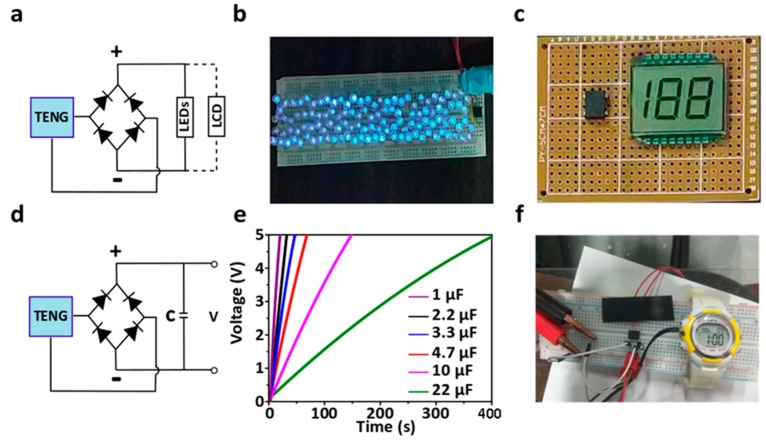
Applications of GS-TENG. (**a**) Equivalent circuit diagram with GS-TENG for powering light emitting diodes (LEDs) and liquid crystal display (LCD). (**b**) More than 120 LEDs connected in series and (**c**) LCD through a rectifying bridge powered by GS-TENG. (**d**) The GS-TENG is connected to a full wave bridge rectifier circuit with capacitor. (**e**) Charging curve of commercial capacitors with different capacitance (1, 2.2, 3.3, 4.7,10 and 22 µF) charged for 5 V.(**f**) 22 µF capacitor being charged by GS-TENG for 5 V and used to power electronic watch.

The practical applications of as-fabricated GS-TENG are demonstrated in Figure 6. The output we obtain will be an alternate current. It is converted to direct current output by a rectifying circuit, as shown in Figure 6a. The as-fabricated GS-TENG can drive more than 120 commercial blue LEDs (Supplementary Video S1) connected in series, as shown in Figure 6b, and it can also power LCD, as shown in Figure 6c, through a rectifier circuit (Supplementary Video S2). The output power generated by the as-fabricated TENG can be stored using a rectifier bridge in a capacitor or battery, as shown in Figure 6d, which can be further used to power some electronic devices. Figure 6e shows the charging curves of capacitors with various capacitance 1 µF, 2.2 µF, 3.3 µF, 4.7µF, 10 µF and 22 µF, under hand tapping. The charging curve of all the capacitors reveals that it can be charged to 5 V through a rectifier circuit within a short time of 18.64 s, 30.59 s, 44.72 s, 64.28 s, 143.61 s and 393.56 s respectively, by gentle hand tapping. Further, for powering some electronic devices, the stored energy in the capacitor 22 µF with 5 V is utilized to power electronic watch, as shown in Figure 6f (Supplementary Video S3). The applications demonstrated are derived from hand tapping on GS-TENG. Thus, the biomechanical energy, mainly human-related kinetic energy, can be harvested effectively to generate electricity.

**Figure 7:**
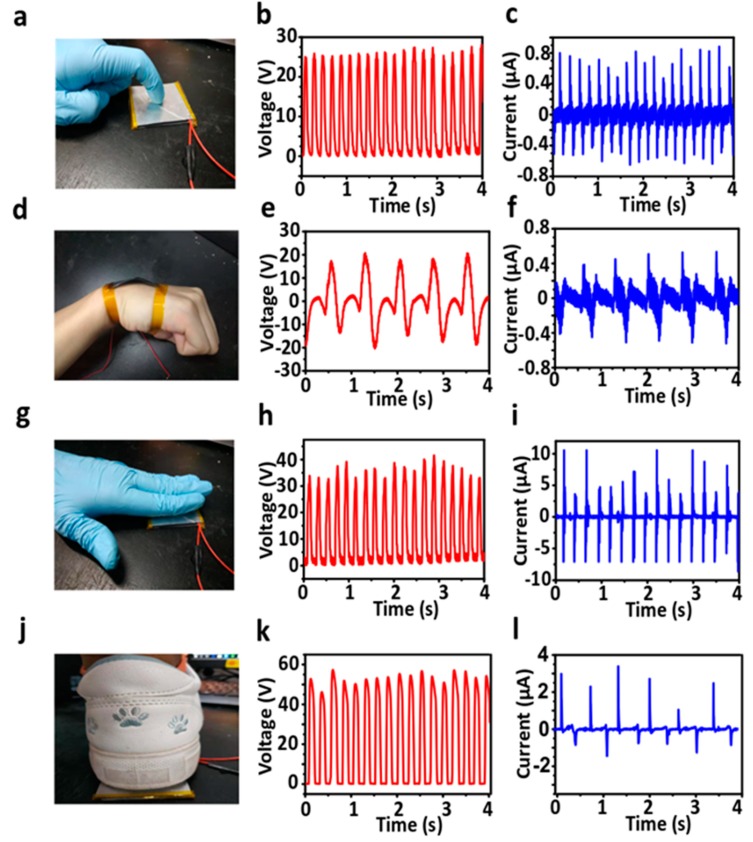
Investigation of energy harvesting from human body movements as an active biomechanical sensor.Optical image of harnessing biomechanical movement and corresponding open-circuit voltage (V_oc_) and short-circuit current(I_sc_) on GS-TENG (**a–c**) finger touch, (**d–f**) wrist movement, (**g–i**) hand tap and (**j–l**) foot stamping respectively.

Additionally, GS-TENG is flexible and can be integrated easily on most of the body parts. As an active wearable electronic device, GS-TENG can detect the body motions. By harvesting biomechanical energy, movements such as finger tapping, wrist movement, hand tapping and foot stepping, onto the as-fabricated GS-TENG are responsible for generating electrical signals. The potential difference generated under each condition depends on whether the GS-TENG is triggered by finger, wrist, wrist or foot movements. As the finger is tapped (Figure 7a), the flexion and extension behaviors from wrist (Figure 7d), hand tapping (Figure 7g) and foot stamping (Figure 7j) periodically results in the electric signals of the corresponding output voltage/current of 26.83 V/14.14 µA, 39.89 V/8.86 µA, 43.84 V/17.52 µA and 55.55 V/4.93 µA respectively, as shown in Figure 7(b/c, e/f, h/i and k/l). Since the output signals produced during each motion state are different in terms of amplitude, number of peaks, peak width, shape and time interval due to these signals depend on the type of activity which contains more information including frequency, pressure and applied force. By distinguishing the output signals, GS-TENG can be used as a self-powered, active biomechanical sensor. With the ease of fabrication, flexibility, eco-friendly, biocompatibility and integration on the human body, our sandpaper-based device will be remarkable to build smart sensors of the next generation and biomechanical energy harvesters [41,42].

## 4. Conclusion

In summary, a novel micro/nano-mesh network sandpaper electrode fabrication process was reported by brush coating of graphite powder. The electrode fabricated is risk-free at the manufacturer and consumer stages, providing durable and disposable features without harming the environment. Moreover, it showed an excellent mechanical stability after integrating with triboelectric layer PTFE, without undergoing any change in electrical property. The as-fabricated GS-TENG is economical, adaptable and can be adopted with any wearable devices to harvest biomechanical energy from the surrounding environment. The maximum peak-to-peak open-circuit voltage, short-circuit current and power density obtained were 36 V, 2.17 µA and 0.941 µW·cm^-2^. The output obtained could be able to charge capacitors and power LEDs, LCD and smart watches by hand tapping. Moreover, GS-TENG acts as a self-powered, active biomechanical sensor by distinguishing the output signals generated from the various human body motions. Thus, sandpaper as a substrate is one of the preferable choices to integrate over cellulose-based paper to enhance the durability of the device, when working with harsh environmental conditions. Since we proved sandpaper’s potential role in electrode fabrication, it could be one of the options to use as a synergistic material in the fabrication of low-cost TENG.

## Supplementary Materials

The following are available online at https://www.mdpi.com/2079-4991/9/11/1585/s1, Figure S1: The relative voltage of GS-TENG for different grit size of the sandpaper, Table S1: The characteristics which make graphite coated sandpaper electrode unique over other metallic based electrodes, Video S1: Powering more than 120 LEDs by GS-TENG. Video S2: Powering LCD by GS-TENG. Video S3: Powering digital watch by GS-TENG.
