# Supplementary material for: Dry-Coated Graphite onto Sandpaper for Triboelectric Nanogenerator as an Active Power Source for Portable Electronics
Source: Nanomaterials (Basel). 2019 Nov 8;9(11):1585. doi: 10.3390/nano9111585 (PMC6915370; doi:10.3390/nano9111585)
Supplement: Supplementary file 1 [file nanomaterials-09-01585-s001.zip › Supporting_Info/Nanomaterials_SM.docx]

**Supplementary Materials**

Smitha Ankanahalli Shankaregowda ^1^, Rumana Farheen Sagade Muktar Ahmed ^2^, Yu Liu ^3,4^, Chandrashekar Bananakere Nanjegowda ^3,4^, Xing Cheng ^3,4^, Srikantaswamy Shivanna ^5,^*, Seeram Ramakrishna ^6^, Zhenfei Yu ^7^, Xiang Zhang ^8,^* and Krishnaveni Sannathammegowda ^2,^*

^1^ Department of Electronics, Yuvaraja’s College, University of Mysore, Mysuru-570005, Karnataka, India; smithamtech@gmail.com (S.A.S)

^2^ Department of Studies in Physics, University of Mysore, Mysuru-570006, Karnataka, India; rumanafarheen1993@gmail.com (R.F.S.M.A)

^3^ Shenzhen Key Laboratory of Nanoimprint Technology, Department of Materials Science and Engineering, Southern University of Science and Technology (SUSTech), Shenzhen 518055, China; liuy3@mail.sustech.edu.cn (Y.L.); chandrashekar@sustech.edu.cn (C.B.N.); chengx@sustc.edu.cn (X.C.)

^4^ Guangdong Provincial Key Laboratory of Energy Materials for Electric Power, Shenzhen 518055, China

^5^ Centre for Materials Science and Technology, Vijnana Bhavan, University of Mysore, Manasagangotri, Mysore 570006, India

^6^ Department of Mechanical Engineering, National University of Singapore, 117576, Singapore; seeram@nus.edu.sg (S.R.)

^7^ School of Materials Science and Engineering, Beijing Institute of Technology, Beijing100081, China; yuzhenfei@bit.edu.cn (Z.Y.)

^8^ School of Mechanical Engineering, Beijing Institute of Technology, Beijing100081, China.

***** Correspondence: [srikantas@hotmail.com(S.S.)](mailto:srikantas@hotmail.com(S.S.)); johnnynash20041579@yahoo.com (X.Z.); sk@physics.uni-mysore.ac.in (K.S.); Tel.: +91-821-410552 (S.S.); +86-135-441-79862 (X.Z.); + 91-821-2419608 (K.S.)

**Supplementary Note: 1**

**Energy conversion efficiency** is defined as the ratio of output electrical energy delivered across the optimal load by the TENG to the input mechanical energy of TENG

**Electrical energy**${(E}_{ele})$

$$E_{ele}=\int_{t_{1}}^{t_{2}} I^{2}R dt$$

$$E_{ele}=R\int_{t_{1}}^{t_{2}} I^{2} dt$$

$E_{ele}$ = 0.2789 µJ

Where, t_1_=1.699 s and t_2_=1.769 s are the time intervals for the positive half cycle

I is output current, R=30 M Ω is optimal load resistance and dt = 0.002 s

**Mechanical energy**

$$E_{mec}=\frac{1}{2}mv^{2}$$

$$=\frac{1}{2} x 1.29 x {10}^{-3} x \left( 0.075 \right)^{2}$$

= 3.62 µJ

Where, m is the mass of movable (upper) layer = 1.29 g

Considering top layer Al/PET is performing simple harmonic oscillations with frequency 4Hz

$$Acceleration={(2\pi f)}^{2} x$$

From acceleration, velocity is derived as

$velocity=\left( 2\pi f \right)x$ = (2 x 3.14 x 4) x 0.003

= 0.075 m/s

Max distance of separation (x) = 0.3 cm

Efficiency can be calculated as

$$\mathrm{Efficiency}\left( \eta\right)=\frac{E_{ele}}{E_{mec}} x 100$$

$$=\frac{0.2789 \mu J}{3.62 \mu J} x 100$$

= 7.7%


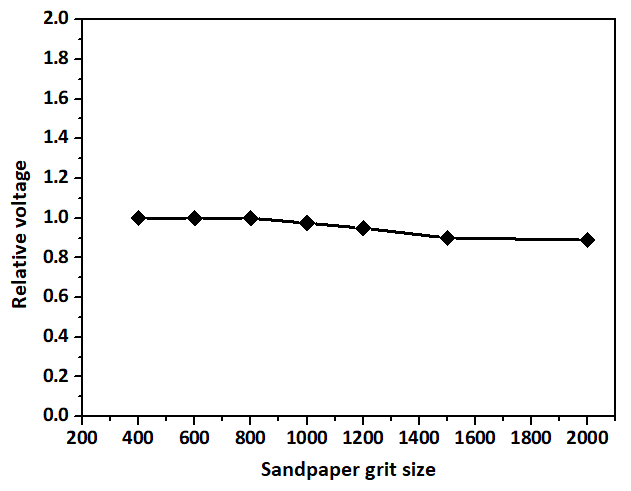


Figure S1: The relative voltage of GS-TENG for different grit size of the sandpaper.

Table S1: The characteristics which make graphite coated sandpaper electrode unique over other metallic based electrodes.

| Characteristics | Graphite coated sandpaper electrode | Other electrodes including Al |
| --- | --- | --- |
| Cost | Cheap | Relatively expensive |
| Manufacturing technique | Facile | Complicated |
| Interaction with atmosphere | Corrosion free | Corrosive |
| Disposable | Yes | No |
| Control of sensing area | Yes | No |
| Availability | Easily available | Available |

As shown in table S1; graphite coated sandpaper electrode provides high surface area due to presence of micro structures, good conductivity ease to use, cheap, commercially available and easily disposable and mechanically rigid. Usually, electrode could not be employed as disposable electrodes due to high cost and their manufacturing methods, but graphite coated sandpaper electrodes are disposal and eco-friendly.

Moreover, Graphite electrodes possess sensitivity compared to metal based electrodes and hence they are widely used in sensors [1]. Also, useful sensors are recognized not only by their cost but the simplicity of fabrication and operation.

**Supplementary Video S1:** Powering more than 120 LEDs by GS-TENG

**Supplementary Video S2:** Powering LCD by GS-TENG

**Supplementary Video S3:** Powering digital watch by GS-TENG

**Reference**

[1] Rana, A. and Kawde, A.N., 2016. Novel Electrochemically Treated Graphite Pencil Electrode Surfaces for the Determination of Trace α‐Naphthol in Water Samples. Journal of the Chinese Chemical Society, 63(8), pp.668–676.
